# Non-disruptive matrix turnover is a conserved feature of biofilm aggregate growth in paradigm pathogenic species

**DOI:** 10.1128/mbio.03935-24

**Published:** 2025-02-21

**Authors:** Courtney Reichhardt, Michael L. Matwichuk, Lincoln T. Lewerke, Holly M. Jacobs, Jing Yan, Matthew R. Parsek

**Affiliations:** 1 Department of Microbiology, University of Washington 7284 , Seattle, Washington, USA; 2 Department of Molecular, Cellular and Developmental Biology, Yale University 5755 , New Haven, Connecticut, USA; Georgia Institute of Technology, Atlanta, Georgia, United States

**Keywords:** biofilms, *Pseudomonas aeruginosa*, *Vibrio cholerae*, CdrA, Psl, exopolysaccharides

## Abstract

Bacteria form multicellular aggregates called biofilms. A crucial component of these aggregates is a protective matrix that holds the community together. Biofilm matrix composition varies depending upon bacterial species but typically includes exopolysaccharides (EPS), proteins, and extracellular DNA. *Pseudomonas aeruginosa* is a model organism for the study of biofilms, and in non-mucoid biofilms, it uses the structurally distinct EPS Psl and Pel, the EPS-binding protein CdrA, and eDNA as key matrix components. An interesting phenomenon that we and others have observed is that the periphery of a biofilm aggregate can be EPS-rich and contain very few cells. In this study, we investigated two possible models of assembly and dynamics of this EPS-rich peripheral region: (i) newly synthesized EPS is inserted and incorporated into the existing EPS-rich region at the periphery during biofilm aggregate growth or (ii) EPS is continuously turned over and newly synthesized EPS is deposited at the outermost edge of the aggregate. Our results support the latter model. Specifically, we observed that new EPS is continually deposited at the aggregate periphery, which is necessary for continued aggregate growth but not aggregate stability. We made similar observations in another paradigm biofilm-forming species, *Vibrio cholerae*. This pattern of deposition raises the question of how EPS is retained. Specifically, for *P. aeruginosa* biofilms, the matrix adhesin CdrA is thought to retain EPS. However, current thinking is that cell-associated CdrA is responsible for this retention, and it is not clear how CdrA might function in the relatively cell-free aggregate periphery. We observed that CdrA is enzymatically degraded during aggregate growth without negatively impacting biofilm stability and that cell-free CdrA can partially maintain aggregation and Psl retention. Overall, this study shows that the matrix of *P. aeruginosa* biofilms undergoes both continuous synthesis of matrix material and matrix turnover to accommodate biofilm aggregate growth and that cell-free matrix can at least partially maintain biofilm aggregation and EPS localization. Furthermore, our similar observations for *V. cholerae* biofilms suggest that our findings may represent basic principles of aggregate assembly in bacteria.

**IMPORTANCE:**

Here, we show that, to accommodate growing cellular biomass, newly produced Psl is deposited over existing Psl at the periphery of biofilm aggregates. We demonstrated that *V. cholerae* employs a similar mechanism with its biofilm matrix EPS, VPS. In addition, we found that the protease LasB is present in the biofilm matrix, resulting in degradation of CdrA to lower molecular weight cell-free forms. We then show that the released forms of CdrA are retained in the matrix and remain functional. Together, our findings support that the *P. aeruginosa* biofilm matrix is dynamic during the course of aggregate growth and that other species may employ similar mechanisms to remodel their matrix.

## INTRODUCTION

Bacteria form multicellular aggregates often referred to as biofilms, in which bacterial cells are tethered together and encased by a biopolymer-rich extracellular matrix (1). Biofilm matrix components structure the aggregate, tethering the bacteria to one another and to surfaces, and in some cases, can serve biochemically active roles, for example as signaling molecules (2) or enzyme inhibitors (3). Biofilm matrix composition varies depending upon the bacterial strain and environmental growth conditions although in general, the matrix is composed of exopolysaccharides (EPS), proteins, and extracellular DNA (1, 4–6). Biofilms cause infections that are difficult to treat (7, 8) since relative to planktonic bacteria, biofilm bacteria are more able to withstand antibiotics (8–10), the host immune system (3, 11, 12), and mechanical clearance (13, 14). The biofilm matrix is a key player in this recalcitrance since when bacteria are aggregated or surface associated, they exhibit protective gene regulation and physiological states (8, 15). In addition, the biofilm matrix prevents the removal of bacteria via shear force (13), and in some instances, the biofilm matrix acts as a physical barrier that impedes antibiotics from reaching the bacteria (9, 10, 16).

*P. aeruginosa* is a paradigm biofilm-forming organism that causes serious and chronic infections (e.g., infections of burns and wounds and lung infections in people with cystic fibrosis) (17, 18). Known components of the *P. aeruginosa* biofilm matrix include three different EPS (Psl, Pel, and alginate), proteins, and extracellular DNA (19–22). By confocal microscopy imaging of *P. aeruginosa* biofilms cultured in microfluidic devices called flow cells, we and others have observed that EPS, specifically, Psl and Pel, localize to a relatively cell-free peripheral region of biofilm aggregates (21, 23, 24). Similar matrix stratification has been observed for *E. coli* macrocolony biofilms (25–27). *P. aeruginosa* strains that are unable to produce the matrix protein CdrA are worse at retaining Psl at the biofilm aggregate periphery (22). CdrA is large (encoded as a 220 kDa protein) and contains a highly repetitive core region (22, 28). It is positively regulated by the intracellular second messenger cyclic di-GMP and is the cargo of the two-partner secretion system encoded by the *cdrAB* operon (22). The pore CdrB is required for export of CdrA from the periplasm to the extracellular space. The periplasmic protease LapG, which is also regulated by c-di-GMP, cleaves CdrA near its C-terminus to release CdrA from the bacterial surface (29, 30). This results in both cell-associated and cell-free forms of CdrA. Furthermore, the presence of the extracellular protease elastase B (LasB) in cell-free culture supernatants results in CdrA degradation (31). The cell-associated form of CdrA promotes hyperaggregation in liquid culture, and release of CdrA from the cell surface or its degradation is anticipated to lead to biofilm disruption (29, 30). Similar adhesin/LapG systems are found in other bacteria (32) including *P. putida* and *P. fluorescens* (33–35) and *Vibrio cholerae* (36). For *P. putida* and *P. fluorescens*, release of the LapA adhesin due to LapG proteolysis is implicated in dispersion (33, 37, 38). In *P. aeruginosa*, dispersion caused by the glycoside hydrolase PslG (which digests Psl) is less efficient in a Δ*lapG* strain background (39). This finding was speculated to be due to the combined impact of retention of CdrA at the bacterial cell-surface and CdrA-Psl interactions being protective against PslG (39). While the cell-associated form of CdrA is clearly important for bacterial aggregation, it has not been determined if the degraded, cell-free forms of CdrA or other similar adhesins contribute to biofilm structure and stability or have another specific function.

Biofilm aggregates are not static. As part of the biofilm lifecycle, biofilm aggregates can expand or lose biomass (40–44). Loss of biofilm biomass can occur through different mechanisms including dispersion, which is an active process in which bacteria escape the biofilm aggregate and return to the planktonic state (45). Dispersion can be triggered by environmental cues, which lead to decreased levels of cyclic-di-GMP and consequently, increased motility and reduced matrix production (33, 37). There is evidence that dispersion requires the biofilm community to produce matrix-degrading enzymes that break apart the matrix to allow bacteria to escape (46). In addition, strategies to prevent or disrupt biofilms include the use of enzymes to degrade biofilm matrix components (47, 48). For example, the glycoside hydrolases PelA and PslG can digest EPS to prevent and disrupt *P. aeruginosa* biofilms (49, 50), and DNase treatment can inhibit or disrupt some biofilms (19, 51). Given the similarities in matrix building blocks across bacterial species such as *P. aeruginosa*, (52)*V. cholerae* (4, 36, 53–57)*, Bacillus subtilis* (58)*,* and others (28, 32, 59, 60) (i.e., all use protein adhesins and EPS to assemble their biofilm matrix), the question is raised if there are also similar mechanisms that control matrix assembly and dynamics.

In this study, we sought to answer how the matrix is remodeled during biofilm growth, and how stable the different matrix components are in the biofilm environment. Specifically, to accommodate growing cellular biomass, the matrix at the aggregate periphery would need to remodel. Thus, we examined the production and retention of the EPS Psl over the course of *P. aeruginosa* biofilm aggregate growth. We observed that newly produced Psl is deposited over existing Psl at the periphery of biofilm aggregates. Next, we demonstrated that *V. cholerae* employs a similar mechanism with its biofilm matrix EPS, VPS (53). For the *P. aeruginosa* matrix protein CdrA to be effective in the matrix at the aggregate periphery, we hypothesized that the cell-free form would be both functional and temporally stable. We identified that LasB is present in the biofilm matrix, resulting in proteolysis of CdrA to lower molecular weight fragments. We provide evidence that proteolysis of CdrA by either LapG or LasB is not detrimental to the biofilm and that degraded, cell-free forms of CdrA are retained in the matrix and can remain functional. Together, our findings support that the *P. aeruginosa* biofilm matrix is dynamic and that paradigm biofilm species may employ similar mechanisms to remodel their matrix during aggregate growth.

## RESULTS

### New Psl is deposited at the aggregate periphery and is required for aggregate expansion

**Fig 1:**
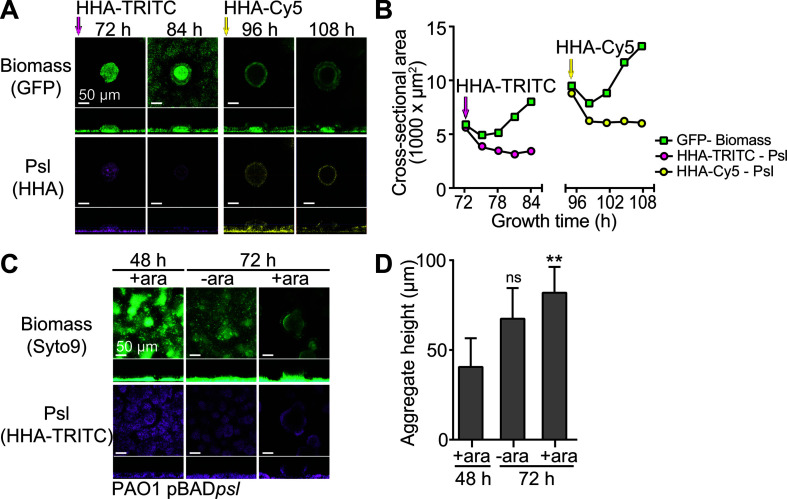
New Psl is deposited at the aggregate periphery and is not required for aggregate maintenance. (**A**) Flow cell biofilms were labeled at different timepoints with fluorescently conjugated Psl-specific lectins and imaged by confocal microscopy. Shown is a typical aggregate observed at this time point. Fluorescence due to GFP is shown in green, HHA-TRITC is shown in magenta, and HHA-Cy5 is shown in yellow. (**B**) The cross-sectional areas of biofilm biomass and Psl observed by microscopy were measured, and the values support that Psl does not migrate to the aggregate periphery but that new Psl is deposited at the aggregate periphery. Data were generated from staining and tracking a single aggregate. (**C**) Flow cell biofilms were grown in which Psl was conditionally expressed via the arabinose-inducible pBAD promoter using the PAO1 pBAD*psl* strain and then imaged by confocal microscopy. Biomass is stained with Syto9 (green), and Psl is stained with HHA-TRITC (magenta). (**D**) The aggregate heights of each growth condition were measured and compared to the aggregate height observed for continuous Psl production (+ara; growth with arabinose) for 48 h. The values support that continuous Psl production is necessary for aggregate growth but is not required for maintaining aggregate height. Data represent the means of results from six images, and error bars indicate standard deviations. The asterisks indicate a significant difference in the aggregate height of aggregates at 72 h compared to aggregates at 48 h (Student’s *t* test; *P* < 0.005), n.s., not statistically significant.

Past studies using confocal microscopy of flow cell-grown *P. aeruginosa* biofilms stained with a fluorophore-conjugated Psl-specific lectin have shown that Psl forms a distinct, peripheral shell that encompasses biofilm aggregates, and this peripheral EPS-rich region is relatively devoid of bacterial cells (21–23, 61). To examine how this Psl shell forms, we performed a pulse-chase labeling experiment of PAO1 GFP^+^ flow cell biofilms using a Psl-specific lectin, Hippeastrum hybrid agglutinin (HHA), conjugated to different fluorophores (either TRITC or Cy5). At 72 h of growth, we applied HHA-TRITC to the biofilms, then used confocal microscopy to monitor fluorescence for 24 h before applying HHA-Cy5 to the biofilms (growth time = 96 h), after which we monitored aggregates for an additional 24 h. We observed that the circumferential footprint of the existing Psl (stained with HHA-TRITC) at the 72 h growth time did not migrate outward with the rest of the biomass at the periphery of the growing aggregate at later time points (Fig. 1A; Fig. S1). However, new Psl (stained with HHA-Cy5) was detected at the aggregate periphery when the aggregates were re-stained at 96 h, and monitored for another 24 h. Again, the circumferential footprint of this signal did not expand to remain at the periphery of the aggregate. These results are depicted in the plot of the area encompassing the lectin staining (TRITC or Cy5) over time compared to the area encompassing the aggregate (GFP) over time (Fig. 1B; Fig. S2). We observed that both the HHA-TRITC and HHA-Cy5 fluorescence decreased over the experiment, which could be due to loss of binding of the Psl-binding lectin, loss of initial Psl from the aggregate, or photobleaching. We also noted an initial decrease in the area encompassing the aggregate (GFP) following lectin staining, which we believe may be due to perturbations during the application of the Psl-binding lectin since this required the media flow to be temporarily stopped. Overall, these results suggested to us that new Psl is deposited over the top of existing Psl at the aggregate periphery to form the Psl shell that has been observed to surround biofilm aggregates. In addition, our findings are also consistent with new Psl being deposited at the periphery and older Psl being gradually lost and suggest that matrix turnover could occur without disruption of the biofilm aggregate.

To determine whether continuous Psl production was necessary for aggregate growth or Psl retention and localization, we performed an experiment in which we conditionally expressed Psl via the arabinose-inducible pBAD promoter using the PAO1 pBAD*psl* strain. For the first 48 h of growth, we included arabinose in the growth medium so that Psl was produced. Biomass was stained with Syto9, and Psl was stained with HHA-TRITC. Using microscopy, we observed biofilm aggregates with associated Psl (Fig. 1C). For the subsequent 24 h of growth, we assessed aggregation and Psl retention and localization in separately cultured biofilms under two conditions: (i) growth in medium that did not include arabinose so that Psl production was halted or (ii) continued growth in medium that included arabinose so that Psl was continuously made. When Psl production was halted by supplying growth medium without arabinose, the biofilms continued to retain Psl, but their growth was stunted (Fig. 1C and D). However, if Psl continued to be produced, we observed that the aggregates continued to grow larger and Psl was detected at the aggregate periphery. This result suggested to us that continuous Psl production is not necessary for the maintenance of biofilms but that new Psl is needed for aggregate expansion.

### *Vibrio cholerae* also deposits EPS at the periphery of growing biofilm aggregates

**Fig 2:**
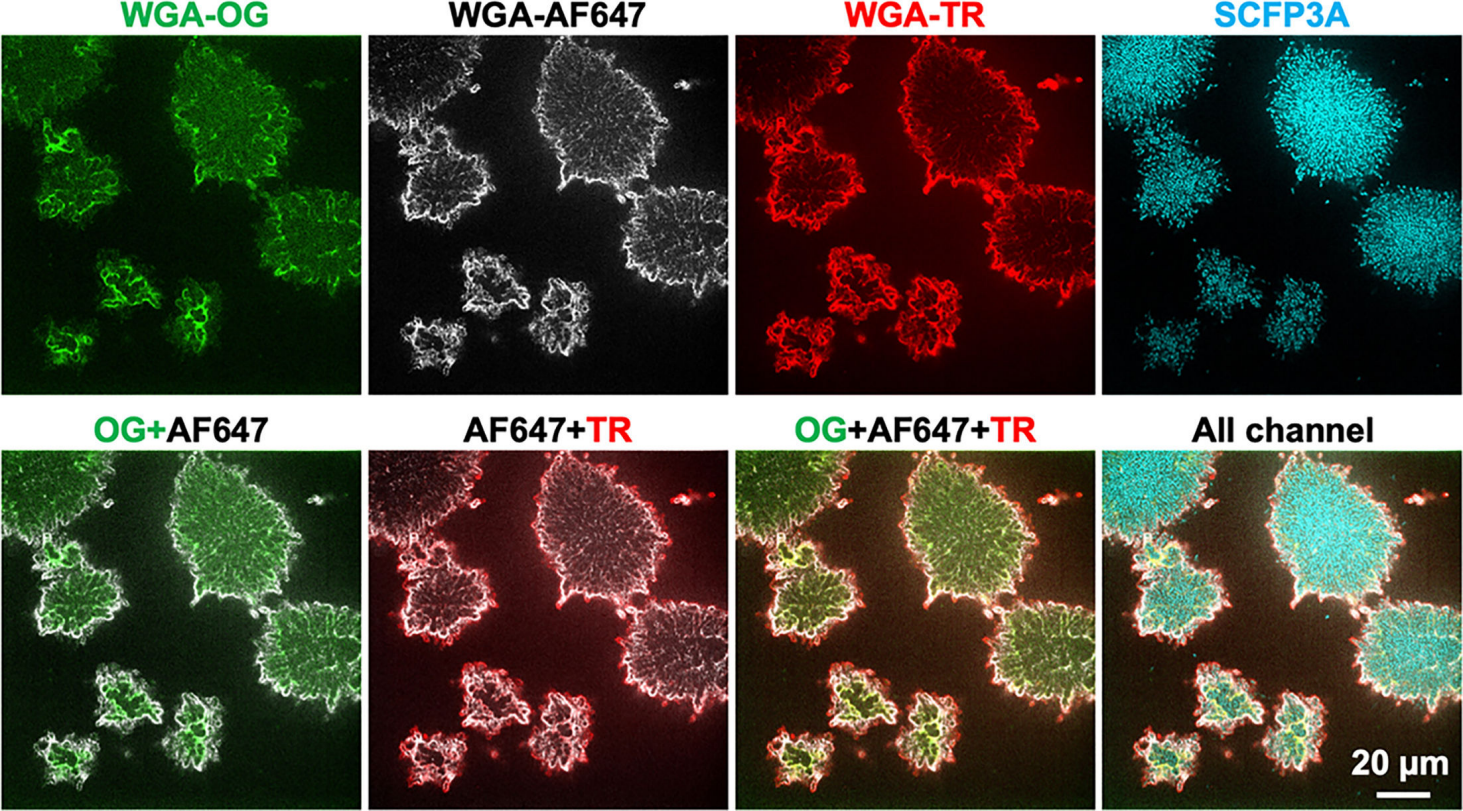
Matrix deposition also is observed in growing, *Vibrio cholerae* biofilm aggregates. Shown are representative cross-sectional images at z = 4 µm for a biofilm from *V. cholerae* cells constitutively expressing SCFP3A. Vibrio polysaccharide (VPS), the major component of *V. cholerae* biofilm, is stained with wheat germ agglutinin (WGA) conjugated with different fluorophores. OG = Oregon Green; AF647 = Alexa Fluor 647; TR = Texas red. The biofilm was first grown from single founder cells in the presence of WGA-OG for the first 27 h, followed by 4 h growth in fresh medium containing WGA-AF647(31 h) and finally another 12 h growth in fresh medium containing WGA-TR (45 h). The top panels show signals from individual stains, while the panels below show different signal overlays. Data were obtained from staining and tracking during a single biological replicate.

We hypothesized that EPS deposition at the periphery of biofilm aggregates may represent a conserved principle for biofilm-forming species. To test this hypothesis, we investigated matrix dynamics using another paradigm biofilm-forming species, *V. cholerae*, which has a well-characterized biofilm matrix, with VPS being the primary EPS in the matrix (55). For these experiments, individual aggregates were grown in the presence of a VPS-binding lectin, wheat germ agglutinin (WGA) conjugated to different fluorophores (OG, AF647, or TR) (54). Specifically, biofilms were cultured with WGA-OG for the first 27 h, followed by 4 h growth in fresh medium containing WGA-AF647, and finally another 12 h of growth in fresh medium containing WGA-TR (Fig. 2). These temporal waves of differential lectin staining produced similar localization patterns to those we observed for *P. aeruginosa*. Application of new lectins resulted in staining at the biofilm aggregate periphery, with little overlap between the other lectins applied at different time points.

### Cell-free forms of CdrA are retained in the biofilm matrix

**Fig 3:**
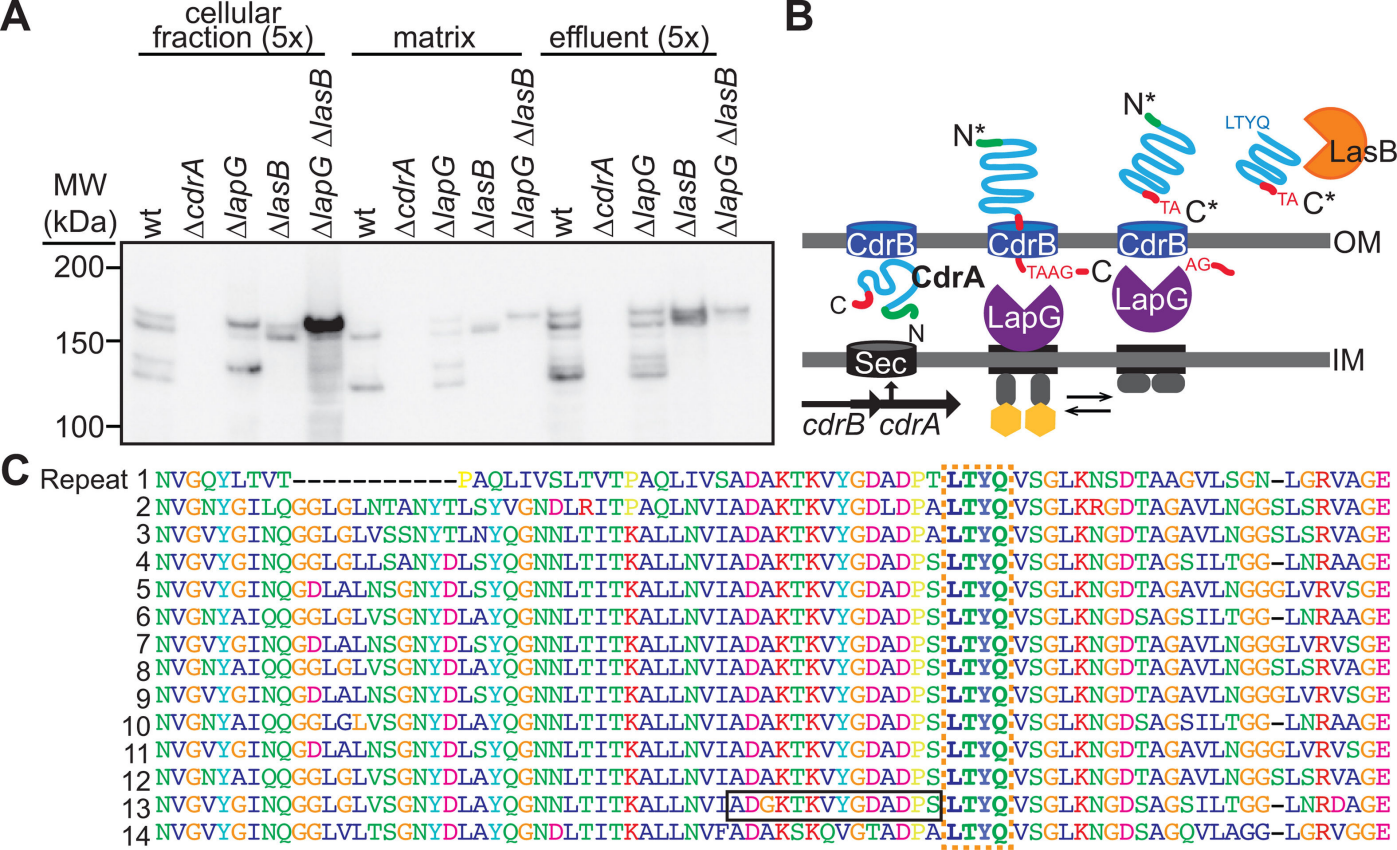
The matrix protein CdrA is degraded in biofilms by at least two proteases. (**A**) Anti-CdrA western blot analysis of tube biofilm fractions of PAO1 and isogenic mutants shows that CdrA is degraded to smaller molecular weight fragments by both LapG and LasB and that CdrA and processed CdrA are retained in the cell-free matrix fraction. (**B**) Schematic representing the secretion and degradation of CdrA by the periplasmic protease LapG and extracellular protease LasB (adapted from references 30, 31 with permission of the publisher). (**C**) CdrA contains 14 imperfect repeats of 81 amino acids. The antigenic peptide for the CdrA-core antibody is indicated by the black box, and the LasB cut site is indicated by the orange dashed box.

The Psl results (Fig. 1) justify investigating the temporal stability of CdrA in the matrix environment and its potential role in the relatively cell-free aggregate periphery. Since we hypothesized that cell-free CdrA would be necessary to help retain Psl in the relatively cell-free, Psl-rich biofilm periphery, we sought to first determine which forms of CdrA—cell-associated or cell-free forms—were present in the biofilm matrix. We compared WT to strains that are missing the ability to release CdrA from the cell surface so primarily have only cell-associated CdrA (Δ*lapG*) and strains that are incapable of LasB proteolysis (Δ*lasB*). To do so, we examined CdrA present in the matrix by western blot analysis (Fig. 3A; Fig. S3). To do this, we use a previously published approach to separate biofilm cells from the matrix (see Materials and Methods and references 28, 32). We noted that in addition to cell-associated CdrA, we observed that extracted biofilm matrix retained cell-free CdrA, including some lower-molecular weight forms of CdrA (the identities of individual bands are depicted in Fig. S3). Based on the existing evidence of CdrA structure and processing, we predicted that these CdrA fragments were CdrA that is cleaved at its C-terminus by LapG (29, 30) (and primarily found as an approximately 150 kDa protein) and at its N-terminus by an unknown mechanism (22) (Fig. 3B). Since the N-terminal fragment of CdrA encompassing residues 1–437 that is cleaved from the cell-free form of CdrA could also be retained in the biofilm, we investigated its function. We found that it alone cannot promote biofilm formation, and instead, this N-terminal region is likely an extended signal sequence for the export of CdrA from the cell (Fig. S4 and S5) (22, 62, 63). From our prior work, we know that the presence of the extracellular protease LasB results in the degradation of purified CdrA (31). Thus, we predicted that CdrA fragments with molecular weights less than approximately 150 kDa resulted from LasB proteolysis (Fig. 3A and B). We systematically examined the biofilm presence of each of these CdrA fragments. Using Δ*lapG* and Δ*lasB* strains, we observed that biofilms of the Δ*lapG* strains had decreased levels of cell-free CdrA and that the Δ*lasB* strains did not generate lower molecular weight fragments of CdrA (Fig. 3B; Fig. S3; Table S1). This supported that cell-free CdrA is primarily generated by LapG, although the presence of CdrA in the matrix of the Δ*lapG* strains suggests that additional mechanisms, such as another protease or cell lysis during biofilm growth, may result in the generation of cell-free CdrA independently of LapG. These results also supported that the smaller CdrA fragments appear to be generated via proteolysis by LasB. In addition, using anti-LasB western blot analysis and a fluorogenic LasB activity assay, we determined that LasB is present in the cell-free matrix and is active (Fig. S6).

We identified where LasB-mediated cleavage of CdrA occurred using N-terminal sequencing of the approximately 125 kDa fragment generated using purified CdrA and LasB-containing, cell-free supernatant preparations (corresponds to Fragment “D” from Fig. S3). We found that the N-terminal sequence of this fragment corresponded to either LTYQ or TYQ. The LTYQ sequence matches what would be expected for a thermolysin-like protease, such as LasB, which typically cleaves peptide bonds at the N-terminal side of hydrophobic amino acids (64, 65). Interestingly, this LasB cut site is repeated throughout the CdrA repeat region (Fig. 3C), which could give rise to the multiple CdrA bands observed in Fig. 3A and previous studies (31). For reference, a single 81-amino acid repeat is approximately 8.1 kDa. The relatively high amount of cell-free CdrA that was retained in the biofilm was a surprise to us, and we sought to determine if these forms of CdrA were functional by evaluating the biofilm-promoting ability of the different fragments of CdrA that arise from degradation. The fragments that we investigated moving forward included the approximately 150 kDa fragment that arises from LapG cleavage (cell-free form), and the approximately 125 kDa fragment that arises from LasB-mediated proteolysis.

### Cell-free CdrA is functional and sufficient for continued biofilm aggregate growth

**Fig 4:**
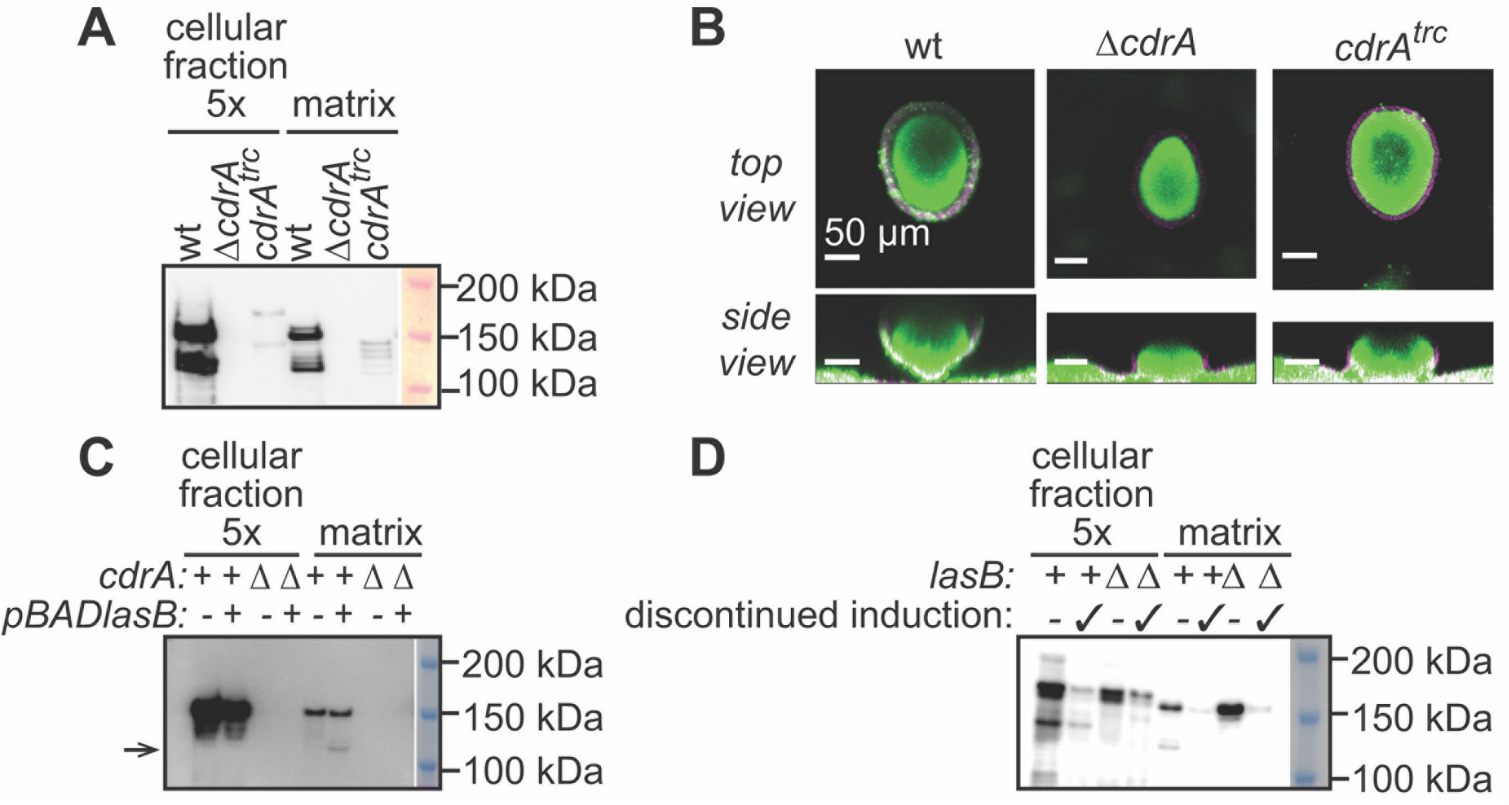
CdrA degradation is not detrimental to biofilm formation. (**A**) Anti-CdrA western blot analysis of cellular and cell-free matrix fractions extracted from tube biofilms of PAO1 (wt), PAO1 Δ*cdrA*, and PAO1 *cdrA^trc^*. (**B**) Representative confocal microscopy images of flow cell biofilms of PAO1 Δ*wspF* (wt), PAO1 Δ*wspF* Δ*cdrA*, and PAO1 Δ*wspF cdrA^trc^*. Biomass is stained with Syto9 (green) and Psl is stained with HHA-TRITC (magenta). The biofilm height of imaged micrographs are as follows: wt: 84 µm; Δ*cdrA*: 48 µm and *cdrA^trc^*: 60 µm. Data shown were obtained from staining and tracking during a single biological replicate. (**C**) Anti-CdrA western blot analysis of cellular and cell-free matrix fractions extracted from tube biofilms in which we conditionally overexpressed *lasB* using a pBAD*lasB* plasmid in the PAO1 Δ*lasB* and PAO1 Δ*cdrA*Δ*lasB* strains. Biofilms grown with strains carrying the pBAD*lasB* plasmid are indicated as *lasB “*+” in the figure, and those with strains carrying the empty vector, pJN105, are indicated as *lasB* “-”. (**D**) Anti-CdrA western blot analysis of cellular and cell-free matrix fractions extracted from tube biofilms in which we conditionally overexpressed *cdrAB* using a pBAD*cdrAB* plasmid in the PAO1 Δ*cdrA* and PAO1 Δ*cdrA*Δ*lasB* strains for the first 48 h of growth. Then biofilms were either harvested at 48 h (-) or induction of *cdrAB* was discontinued for the subsequent 24 h and then the biofilms were harvested (indicated with a checkmark).

To test whether cell-free CdrA alone is functional, we engineered a construct of CdrA that was truncated at its C-terminus at residue 1972 (CdrA^trc^) such that it is always released from the cell surface. This is likely because CdrA^trc^ is missing the cysteine residues thought to form a cysteine hook, anchoring CdrA to CdrB (22, 30). First, by western blot analysis using the CdrA-core antibody, we investigated whether CdrA^trc^ was retained in the matrix of tube biofilms of PAO1 (Fig. 4A). We observed that CdrA^trc^ is retained in the matrix, albeit at lower levels than wt CdrA. This lower amount of CdrA^trc^ could be due to increased degradation during cellular export or to decreased retention in the biofilm matrix. Next, we imaged flow cell biofilms of PAO1 Δ*wspF* to determine if CdrA^trc^ was able to promote biofilm aggregation and retention of Psl similarly to wt CdrA. We used the PAO1 Δ*wspF* strain since the impact of CdrA on biofilm structure is most evident in this strain (22). As previously observed, the matrix overproducer PAO1 Δ*wspF* expressing wt CdrA assembles as robust aggregates with a tightly associated zone of Psl-staining material, and the isogenic PAO1 Δ*wspF*Δ*cdrA* strain forms aggregates that are shorter (22) (Fig. 4B). This is thought to be because CdrA binds to Psl to promote the formation of aggregates that withstand shear force better than aggregates with Psl alone (22, 31). Aggregates formed by PAO1 Δ*wspF cdrA^trc^* formed aggregates with a tightly associated Psl shell but intermediate height. This result contrasted with our finding that overexpression of *cdrA^trc^* does not promote liquid-culture CdrA-dependent aggregation (not shown). We additionally monitored the formation of the flow cell aggregates over time (Fig. S7). By 48 h of growth, PAO1 Δ*wspF* formed distinct aggregates that extended into the lumen of the flow cell. By contrast, biofilm aggregates formed by PAO1 Δ*wspF*Δ*cdrA* remained shorter, even after 96 h. Meanwhile, for PAO1 Δ*wspF cdrA^trc^*, aggregate growth outpaced the Δ*cdrA* strain, though their ability to form tall aggregates was delayed relative to PAO1 Δ*wspF*. The delay was not due to CdrA^trc^ causing a defect in the initial attachment to the flow cell coverslip, which we investigated using strains carrying either the pBAD*cdrAB* or the pBAD*cdrA^trc^B* plasmid (Fig. S8). Overall, these results suggest that cell-free CdrA is retained in the matrix where it can support aggregate stability through Psl retention.

Since we observed that LasB mediates the degradation of CdrA, we sought to determine whether LasB activity impacted CdrA and its degraded forms in the biofilm matrix. To test this, we conditionally overexpressed *lasB* using a pBAD*lasB* plasmid in the PAO1 Δ*lasB* and PAO1 Δ*cdrA*Δ*lasB* strain backgrounds. We then monitored CdrA proteolysis and retention in the biofilm cell-associated fraction and cell-free matrix fraction relative to the degradation that occurred when the strains carried an empty vector, pJN105 (Fig. 4C; Fig. S9). LasB is important for proteolyzing proteins to amino acids that can be used as nutrients by *P. aeruginosa* (66). As such, for this experiment, the biofilms were cultured in a casamino acid medium so that biofilms expressing LasB would not have a growth advantage compared to Δ*lasB* strains, although we did not observe a LasB-dependent growth defect for planktonic cultures grown in either LB or casamino acid medium (Fig. S10). For the first 48 h of biofilm growth, we did not supply arabinose so biofilms were not making LasB and were able to establish independently of LasB. Then we compared the retention of CdrA in the cases where arabinose was supplied or not for the subsequent 48 h. As expected, in the case where expression of *lasB* was induced, we observed the appearance of a second, lower molecular weight band of CdrA as indicated by the arrow in Fig. 4C. Both the approximately 150 kDa and 125 kDa CdrA bands were retained in the biofilm matrix. We compared the CdrA band intensities in the matrix fractions of PAO1 Δ*lasB* pJN105 versus PAO1Δ*lasB* pBAD*lasB*, and we found that expression of *lasB* did not result in a decrease in CdrA retention (Fig. 4C; Fig. S9) (*n* = 3; *P* = 0.24). These results support that the expression of LasB results in proteolysis of CdrA to a lower molecular weight fragment, but that this form is retained in the biofilm matrix and remains functional.

Since LasB activity did not result in the loss of CdrA from the biofilm matrix, we wondered whether continuous CdrA production was necessary for aggregate growth and maintenance. To test this, we performed an experiment in which we conditionally overexpressed *cdrA* using a pBAD*cdrAB* plasmid in the PAO1 Δ*cdrA* and PAO1 Δ*cdrA*Δ*lasB* strains and then used anti-CdrA western blot analysis to evaluate if retention of CdrA varied (Fig. 4D). For the first 48 h of biofilm growth, we supplied arabinose so that biofilms were making CdrA. We compared the retention of CdrA in biofilms that were harvested at 48 h to those that were harvested after an additional 24 h of growth without arabinose. CdrA remained following growth without arabinose, but at lower relative amounts compared to the initial 48 h of biofilm growth with a continuous supply of arabinose in the growth medium (for PAO1 Δ*cdrA* pBAD*cdrAB*, approximate 6.6-fold decrease; *n* = 3; *P* < 0.05). As in the other experiments described in this study, in the case where *lasB* was present, we observed the appearance of a second, lower molecular weight band of CdrA. Both the approximately 150 kDa and 125 kDa CdrA bands were retained in the biofilm matrix. We compared the CdrA band intensities in the matrix fractions following cessation of induction of *cdrAB* in biofilms formed by PAO1 Δ*cdrA* pBAD*cdrAB* vs. PAO1 Δ*cdrA*Δ*lasB* pBAD*cdrA*B, and we noted that the presence of *lasB* did not result in a decrease in matrix retention of CdrA (Fig. 4D; Fig. S11) (*n* = 3; *P* = 0.56). These results support that continuous production of CdrA is not necessary for its retention in the biofilm matrix.

## DISCUSSION

We show in this study that the matrix of *P. aeruginosa* biofilms undergoes both continuous synthesis of matrix material and matrix turnover to accommodate an expanding biofilm aggregate. Evidence suggests that the EPS component of nonmucoid strains localizes to a discrete zone surrounding growing biofilm aggregates. Although aggregates grow and expand robustly, this zone of matrix EPS remains at the aggregate periphery and at fairly uniform width. Two different ways to maintain a discrete amount of matrix accumulation include (i) low levels of matrix production with minimal matrix loss over the course of biofilm development or (ii) high matrix loss, which is offset by *de novo* matrix production. To explore these possibilities, we first examined the importance of Psl for continued aggregate expansion. Newly produced Psl is deposited over existing Psl at the periphery of biofilm aggregates as the aggregates expand, supporting the notion that *P. aeruginosa* biofilm matrix is dynamic, with both synthesis of new matrix material and degradation of existing matrix being important. We observed a similar phenomenon for VPS in *V. cholerae* biofilms. Given its importance in retaining Psl in *P. aeruginosa* biofilms, we next focused on the matrix structural protein, CdrA. We showed that CdrA cleavage occurs through two proteases, LasB and LapG. LasB is a secreted protease, found associated with biofilm matrix material. We also show that proteolyzed forms of CdrA are retained in the biofilm and remain functional. Overall, we found that the production of either new Psl or new CdrA is not required for aggregate maintenance, which suggests that only low levels of matrix production are required to offset minimal matrix loss over the course of biofilm development. We provide evidence that the biofilm matrix of biofilm aggregates is not static and undergoes remodeling.

Our observations of Psl production and retention during biofilm aggregate growth are consistent with previous observations (67), and Colvin et al. previously observed a similar phenomenon for Pel showing that continuous production of Pel was not necessary to maintain the aggregate (68). Our finding that new Psl is deposited at the periphery of the biofilm aggregate is at odds with a previous study that utilized a *psl* transcriptional reporter and observed *psl* transcription within the interior of aggregates (69). However, we speculate that the peripheral localization of newly deposited Psl could be driven by metabolic or signaling pathways because bacteria at the aggregate periphery have increased metabolism relative to those at the interior since the interior of the aggregate is a poorer nutrient, lower oxygen environment (70). Furthermore, our observation that newly added lectin only stains the periphery and fails to stain the region in the interior of aggregates corresponding to previous lectin staining supports our model that new Psl is deposited at the periphery and previously produced Psl is degraded in the aggregate interior (Fig. 1A and B). In addition, the peripheral localization of Psl coincides with the spatial localization of c-di-GMP-high cells that were observed for mature *P. aeruginosa* biofilm aggregates using a c-di-GMP responsive reporter plasmid (71). Such c-di-GMP-driven local heterogeneity of matrix synthesis was observed for *E. coli* macrocolony biofilms (25, 72). This finding is interesting since c-di-GMP positively regulates many biofilm genes. Alternatively, the location of *psl* transcription may not directly correlate with where Psl exopolysaccharide is retained in a biofilm aggregate.

The importance of Psl at the relatively cell-free periphery raises the question of how Psl remains associated with the aggregate. Thus, we investigated the degradation of CdrA and evaluated whether cell-free forms of CdrA are retained in the matrix and potentially contribute to aggregate growth. We observed that CdrA is proteolyzed by the periplasmic protease LapG and the extracellular protease LasB, which is retained and active in the biofilm matrix (Fig. 3). LasB could be retained in the matrix via direct interactions with either CdrA or Psl, which we are investigating in future studies. Different forms of CdrA are retained in the biofilm and can partially promote the ability of biofilm aggregates to maintain a peripherally localized Psl “shell” and grow into the lumen of the flow cell (Fig. 1). These results also suggest that despite CdrA being released from the cell surface and further degraded by LasB, CdrA appears to be fairly stable in the matrix and LasB activity only minimally depletes CdrA from the biofilm. This stability likely arises in part due to the interactions between CdrA and Psl, which we previously showed protect CdrA from LasB proteolysis. The N-terminal region of CdrA (residues 1–437) alone cannot promote aggregation or biofilm formation, and instead, our results support that this region is an elongated signal sequence similar to what has been observed for other adhesins (62, 63). Overall, our results suggest that CdrA proteolysis does not result in high levels of loss from the biofilm matrix. Based on these results, we have developed a model in which cell-associated matrix is important for early aggregate maturation, driving mechanical cell-cell interactions which are key to early biofilm architecture (42, 43), and cell-free and degraded matrix components are sufficient for aggregation at later stages of biofilm development. We are still continuing to test and refine this model, and it would be interesting to explore whether cell-free and/or degraded matrix components in *P. aeruginosa* biofilms serve as a public good or if they are privatized, similar to what was observed for the matrix *of B. subtilis* biofilms (73). These results, together with our Psl observations, suggest that matrix turnover is a normal aspect of mature biofilm physiology and does not necessarily precede biofilm aggregate disassembly.

As aggregate formation and expansion are key features of biofilm development for a number of different species (44, 74–77), we predict that the dynamics of matrix synthesis and turnover we observe here may translate to other biofilm-producing species, and indeed this is what we observed for biofilms formed by *V. cholerae*. To investigate whether EPS deposition at the periphery of a growing aggregate might be a general feature of bacterial biofilm formation, we investigated temporal EPS distribution in another well-studied species, *V. cholerae*. EPS staining experiments demonstrated that *V. cholerae* appears to layer new EPS at the aggregate periphery during the course of growth. Further research is necessary to reveal the molecular details that lead to the observed pattern. These findings support the idea that matrix dynamics as it relates to aggregate growth may be conserved among species. It will be interesting to investigate this concept further to determine if similar molecular mechanisms that underlie this observation are similarly conserved across species.

As part of the biofilm lifecycle, biofilm aggregates can change in size, growing or losing biomass. This is anticipated to require connections in the biofilm matrix to be broken and new connections to be formed. One way that this remodeling could be achieved is through enzymatic degradation of biofilm matrix components, such as the proteolysis of CdrA by LapG and LasB. In addition to permitting growth, matrix remodeling resulting in localized changes in composition or matrix interactions may give rise to the spatially heterogeneous properties such as viscosity that have been previously observed (78). The remodeling of the biofilm matrix is reminiscent of the remodeling of the eukaryotic extracellular matrix (ECM) that occurs during growth and in response to environmental cues (79). In this study, we observed the retention of LasB, which is an enzyme with multiple functionalities. Similarly, eukaryotic ECM harbors remodeling enzymes, which are typically metalloproteinases, and many have additional essential functions (80). Healthy tissue requires balanced ECM synthesis and degradation. As such, eukaryotic ECM remodeling is tightly controlled at both the transcriptional and posttranscriptional levels, including by specific localization of proteinases, and dysregulation of eukaryotic ECM remodeling can result in disease states such as tumorigenesis or fibrosis. In addition, in some cases, the ECM fragments that arise during the remodeling process are functional and can play a role in cell proliferation or inflammation. In summary, similar to eukaryotic ECM remodeling, biofilm matrix remodeling may serve as an intermediate way that cell-cell and cell-matrix interactions are modulated which is distinct from the more extreme matrix disruption that occurs during dispersal or disassembly.

## MATERIALS AND METHODS

### Bacterial strains, media, and growth conditions

Planktonic cultures were routinely grown on Lysogeny broth (LB) medium at 37 °C with constant shaking (225 rpm) unless indicated otherwise. *P. aeruginosa* PAO1 strains and *Escherichia coli* strains used for mutant construction were cultured in LB broth at 37 °C. For selection and maintenance of plasmids, antibiotics were used at the following concentrations: 100 µg/mL carbenicillin and 10 µg/mL gentamicin for *E. coli* and 300 µg/mL carbenicillin and 30 µg/mL gentamicin for *P. aeruginosa*. All strains are listed in Table S2. A growth curve was performed in LB and casamino acid medium for select isolates (Fig. S10). Mutants were generated using the previously published protocol for allelic replacement in *P. aeruginosa* (81). The *V. cholerae* strain used in this study was a derivative of the wild-type *V. cholerae* O1 biovar El Tor strain C6706str2. The rugose strain background harbors a missense mutation in the *vpvC* gene (*vpvC*^W240R^) that elevates intracellular c-di-GMP levels. A constitutively expressed SCFP3A was genetically engineered into this strain using the natural transformation (MuGENT) method (82). *V. cholerae* was grown overnight in lysogenic broth (LB) at 37°C with shaking. 1× M9 salts were filter sterilized and supplemented with 2 mM MgSO_4_ and 100 µM CaCl_2_ (abbreviated as M9 medium below). Biofilm growth was performed in a M9 medium supplemented with 0.5% glucose.

### Flow cell biofilms and confocal microscopy

Bacterial strains were grown in LB until the mid-log phase and then diluted in 1% LB to a final OD_600_ of 0.01. Flow cell chambers were inoculated with the diluted cultures and incubated inverted for 1  h before initiation of flow. Biofilms were continuously supplied with fresh 1% LB and were grown at room temperature. Psl was stained with the lectin HHA conjugated to a fluorophore (100 µg/mL; GlycoMatrix), which was allowed to interact with the biofilm for 15 min and then washed for 10 min prior to imaging. A Zeiss LSM 800 scanning confocal laser microscope and a Zeiss LSM 510 confocal laser scanning microscope were used to image the *P. aeruginosa* biofilms. *V. cholerae* biofilms were imaged with a spinning disk confocal microscope (Nikon Ti2-E connected to Yokogawa W1) using a 100× oil immersion objective (numerical aperture = 1.35). Variations in growth conditions, imaging, and analysis are described below:

For the pBAD*cdrAB* biofilms (Fig. S4C), the growth medium was supplemented with 1% (wt/vol) L-arabinose and grown for 96 h. The biomass was stained with 2.5 µM Syto 9 (Molecular Probes) to visualize the entire biomass before imaging. Images were collected using a 20× objective. Images were processed using Volocity software (Improvision). The brightness and contrast of microscopy images were adjusted linearly and identically for all images in Photoshop.

For the PAO1 Δ*wspF*, PAO1 Δ*wspF*Δ*cdrA*, and PAO1 Δ*wspF cdrA^trc^* flow cell biofilms (Fig. 4B; Fig. S7), all strains constitutively expressed GFP via a chromosomal insertion [Tn7 Gm::P(A1/04/03)::GFP]. Images were collected at 24 h, 48 h, 72 h, and 96 h. At 96 h, the fluorescent lectin HHA-TRITC (100 µg/mL) was used to stain Psl and was allowed to interact with the biofilm for 15 min prior to washing and imaging. Images were collected using a 40× objective. Images were processed using Volocity software (Improvision). The brightness and contrast of microscopy images were adjusted linearly and identically for all images in Photoshop.

For the time-lapse microscopy of PAO1 Tn7 Gm::P(A1/04/03)::GFP biofilms (Fig. 1A; Fig.S2), Psl was stained with HHA-TRITC at 72 h. Confocal images were then captured using a 20× objective every hour for 12 h. Then at 96 h, Psl was stained with HHA-Cy5. Confocal images were then captured every hour for an additional 12 h. To determine changes in the cross-sectional areas of biomass (GFP) and Psl (HHA-TRITC or -Cy5), images of the cross-section at 28.16 µm (72 to 86 h time-course) or 34.56 µm (96 to 108 h time-course) from the coverslip were identified using Zen imaging software (Zeiss) and exported. The cross-sectional area was measured in Fiji (83) after spatial calibration by using the “polygon selections” tool to trace the circumference of the fluorescent area corresponding to either the biomass (GFP) or Psl (HHA-TRITC or -Cy5). The brightness and contrast of microscopy images were adjusted linearly and identically for all images in Photoshop.

For the PAO1 Δ*psl pBADpsl* flow cell biofilms (Fig. 1B), three separate biofilms were grown and imaged as follows: (i) cultured for 48 h in growth medium that was supplemented with 0.05% (wt/vol) L-arabinose and imaged at 48 h; (ii) cultured for 48 h in growth medium that was supplemented with 0.05% (wt/vol) L-arabinose, then cultured without arabinose for an additional 24 h, and imaged at 72 h; and (iii) cultured for 72 h in growth medium that was supplemented with 0.05% (wt/vol) L-arabinose and imaged at 72 h. The biomass was stained with 2.5  µM Syto 9 (Molecular Probes) to visualize the entire biomass, and Psl was stained with HHA-TRITC. Images were collected using a 20× objective. To determine the aggregate height, six images from each condition were analyzed using Fiji software (83). The brightness and contrast of microscopy images were adjusted linearly and identically for all images in Photoshop.

The biofilm growth procedure for *V. cholerae* was previously described. Briefly, overnight cultures of the strain were grown at 37°C with shaking in 1.5 mL LB. Fifty microliters from each culture was used to inoculate 1.5 mL of M9 medium supplemented with 0.5% glucose and grown at 30°C with shaking until the OD_600_ was between 0.1 and 0.3. The cultures were then diluted to an OD_600_ ≅ 0.001. One hundred microliters of the regrown culture was aliquoted into the wells of a 96-well plate with a glass bottom (MatTek P96G-1.5-5-F) and incubated at 30°C for 30 minutes. The wells were then washed twice with M9 medium and replaced with M9 medium with 0.5% glucose and 0.5 mg/mL bovine serum albumin (BSA), plus fluorescently labeled WGA. The concentration of all dye solutions for staining *V. cholerae* biofilms was 4 µg/mL. A 445 nm laser excitation was used to observe the *V. cholerae* cells, a 488 nm laser for WGA-Oregon Green, a 594 nm laser for WGA-Texas red, and a 640 nm laser for WAG-AF647, with the corresponding filters. The images were captured with a sCMOS camera (Photometrics Prime BSI) at a *z*-step size of 0.5 µm, with 2 by 2 pixel binning.

### Tube biofilms

Tube biofilms were grown as previously described (61). Briefly, silicone tubes were inoculated with a suspension at an OD_600_ of 0.1 and grown at room temperature using 1% LB at a flow rate of 10 mL/h at room temperature for 5 days. At the end of the experiment, 1 mL of effluent was collected. Then flow was stopped, the inlet was blocked and the outlet was disconnected. The contents of the tubes were removed, and the material adhered to the tube walls was physically removed using the handle of an L-shaped loop. To remove the biofilm matrix from the bacterial cells, the biofilm sample was vortexed for 1 min with 0.5 mL of 1 mm glass beads, and the sample was removed with an 18-gauge syringe. The optical density (OD_600_) of the biofilm samples was determined and normalized to 1 mL of OD_600_ 0.75 in 1× PBS. The biofilm sample was centrifuged for 20 min at 7,000 × *g*. The supernatant (“matrix fraction”) was removed and stored at 4°C. The cell pellet was washed in 1 mL of 1× PBS. The cell pellet was then resuspended in 200 µL of 1× PBS, sonicated for 30 s, centrifuged for 2 min at 16,000 × *g*, and the supernatant was saved as the “cell-associated” fraction. The effluent fraction was normalized by volume relative to the biofilm fraction that was normalized based on OD_600_, flash frozen, lyophilized, and resuspended to be fivefold concentrated. Variations in conditions are as follows: the pBAD*lasB* tube biofilms (Fig. 4C; Fig.S9) were grown in 1% (wt/vol) casamino acid (CAA) medium for 48 h, and then in 1% (wt/vol) CAA supplemented with 0.1% (wt/vol) L-arabinose for an additional 48 h. Biofilms were harvested at 96 h. The pBAD*cdrAB* tube biofilms (Fig. 4D; Fig.S11) were grown in 1% LB supplemented with 0.1% L-arabinose for the first 48 h of growth, and then in 1% LB (without arabinose) for an additional 24 h of growth.

### Western blot analysis

Protein gel electrophoresis was carried out using 3%–8% XT Tris-acetate gels (Criterion) for analysis of the core region of CdrA, and 4%–20% TGX Tris-glycine gels (Criterion) for the analysis of the N-terminal region and LasB. CdrA was transferred to 0.2-µm-pore-size polyvinylidene difluoride (PDVF) transfer membrane (Bio-Rad) using Tris-glycine transfer buffer with 20% methanol, and LasB was transferred to 0.2-µm-pore-size nitrocellulose transfer membrane using CAPS transfer buffer with 20% methanol. The primary antibodies were diluted in 1% milk–Tris-buffered saline with Tween 20 (TBST) as follows: the primary CdrA-core antibody (GenScript; raised against CADGKTKVYGDADPS) was diluted to 1/10,000 for planktonically grown samples and 1/100 for analysis of tube biofilm samples; the primary CdrA-N-terminal region antibody (GenScript; raised against CGDFQGRGELPRAKN) was adsorbed against PAO1 Δ*cdrA* cell lysates and diluted to 1/1,000; and the primary LasB antibody (GenScript; raised against AEAGGPGGNQKIGKC) was diluted to 1/1,000. Horseradish peroxidase (HRP)-conjugated goat anti-rabbit antibody (Invitrogen) was used as the secondary antibody. If not specified, the CdrA-core antibody was used in the anti-CdrA western blot analysis. Detection was performed with SuperSignal West Pico chemiluminescent substrate (Thermo Scientific).

### LasB activity assay

The LasB activity present in the biofilm matrix from tube biofilms was determined using a variation of the fluorogenic activity assay previously described (65). Aliquots of 200 µL of either undiluted cell-free biofilm matrix or 1:100 diluted cell-free supernatant preparations were added to the wells of a 96-well microtiter plate (Corning Costar, black with clear bottom). A 22 mM stock of the fluorogenic substrate was prepared by suspending 5 mg of Abz-Ala-Gly-Leu-Ala-p-nitro-benzyl-amide (MW = 583.65; Peptides International [SAG-3905-PI]) in 390 µL of dimethylformamide (DMF). Immediately before measuring fluorescence, the fluorogenic substrate was added to the microtiter plate wells to a final concentration of 220 µM. To do so, 2 µL of 22 mM fluorogenic substrate stock was added to each well containing 200 µL of supernatant. To no-substrate control wells, 2 µL of DMF was added so that the final concentration of DMF in every well is 1% (vol/vol). The fluorescence was measured every minute for 1 h (λ_ex_ *=* 340 nm; λ_em_ *=* 415 nm) using a plate reader (BMG Labtech CLARIOstar Plus).

### Edman degradation

Proteolysis of CdrA was carried out as previously described by incubating purified CdrA with cell-free supernatants from stationary-phase cultures of strain PAO1 Δ*wspF* Δ*cdrA* Δ*EPS* at 37°C for 16  h. Protein gel electrophoresis was carried out using 3%–8% XT Tris-acetate gels (Criterion), and samples were transferred to a polyvinylidene difluoride (PVDF) membrane using CAPS transfer buffer. Edman degradation was performed by the UC Davis Proteomic Core using an ABI Procise sequencer. For each residue/cycle, the free N terminus of each immobilized protein was coupled with phenyl isothiocyanate, cleaved with trifluoroacetic acid, and then converted to the more stable phenylthiohydantion amino acid derivative, which was separated by reverse-phase chromatography and detected by UV-visible spectrophotometry.

### Aggregation assays

Stationary-phase cultures were diluted 20-fold into LB medium supplied with 1% (wt/vol) L-arabinose and 300 µg/mL carbenicillin. Cultures were grown in triplicate at 37°C, with shaking at 225 rpm, for 2 h 15 min. Aggregation was evaluated by visual assessment and the measurement of the optical density (OD_600_).

### Crystal violet assay

Static biofilm formation was assessed using the crystal violet assay as previously described (22). Static biofilms were cultured in Nunc Bacti 96-well microtiter plates using Vogel-Bonner minimal medium (VBMM) supplemented with 1% (wt/vol) L-arabinose and 300 µg/mL carbenicillin. Cultures were incubated statically for 20 h at 37°C before nonadherent biomass was removed and the crystal violet assay performed.

### Attachment assay

Cultures were grown overnight at 37°C with shaking from a single colony in 4 mL of LB supplemented with 300 µg/mL carbenicillin. Overnight cultures were vortexed and back-diluted 1:20 in 4 mL of LB supplemented with 300 µg/mL carbenicillin and 1% (wt/vol) L-arabinose. Bacteria were grown to mid-log (OD_600_ 0.4–0.8) at 37°C with shaking and then were vortexed and back-diluted to a final OD_600_ of 0.1 in 1% LB supplemented with 1% (wt/vol) L-arabinose (no antibiotics). Due to visible aggregation in the pBAD*cdrAB* strain, the OD_600_ for this strain was based on that of the pMJT-1 mid-log culture. Back-diluted cultures were passed through an 18-gauge syringe and used to inoculate flow chambers, which were kept inverted (coverslip side down) for 10 minutes to allow cells to attach before induction of flow. Fresh media was used to wash non-attached cells by flow at 40 mL per hour for 20 minutes. Bacteria were stained with 2.5  µM Syto9 for 15 min. The flow was then reduced to a final constant flow rate of 3 mL per hour, and bacteria were imaged immediately on a Zeiss LSM 800 scanning confocal laser microscope. For every strain, 5 fields of view using identical microscope settings to image GFP fluorescence and Brightfield across all experiments. Images were analyzed using Volocity software (Improvision), and data analysis was performed in GraphPad.

## SUPPLEMENTAL MATERIAL

Supplemental material mbio.03935-24-s0001.pdf Supplemental figures and tables.

ASM does not own the copyrights to Supplemental Material that may be linked to, or accessed through, an article. The authors have granted ASM a non-exclusive, world-wide license to publish the Supplemental Material files. Please contact the corresponding author directly for reuse.
