# Supplementary material for: Non-disruptive matrix turnover is a conserved feature of biofilm aggregate growth in paradigm pathogenic species
Source: mBio. 2025 Feb 21;16(3):e03935-24. doi: 10.1128/mbio.03935-24 (PMC11898600; doi:10.1128/mbio.03935-24)
Supplement: Supplemental material — Supplemental figures and tables. [file mbio.03935-24-s0001.pdf]

SUPPLEMENTAL

A B C

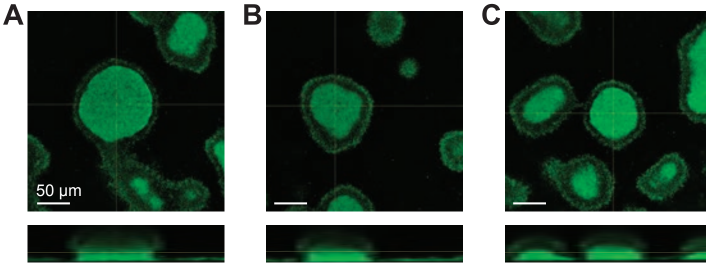

Supplemental Fig. 1. Representative images of flow cell biofilms of PAO1 at 96 h. The aggregate shown in **(A)** is 61 μm tall and 136 μm across; the aggregate shown in **(B)** is 49 μm tall and 103 μm across; and the aggregate shown in **(C)** is 45 μm tall and 100 μm across. These values are comparable to those of the aggregate shown in **Fig 1A**, which at the 96-h timepoint is 59 μm tall and 108 μm across. Fluorescence due to constitutive expression of GFP is shown in green.

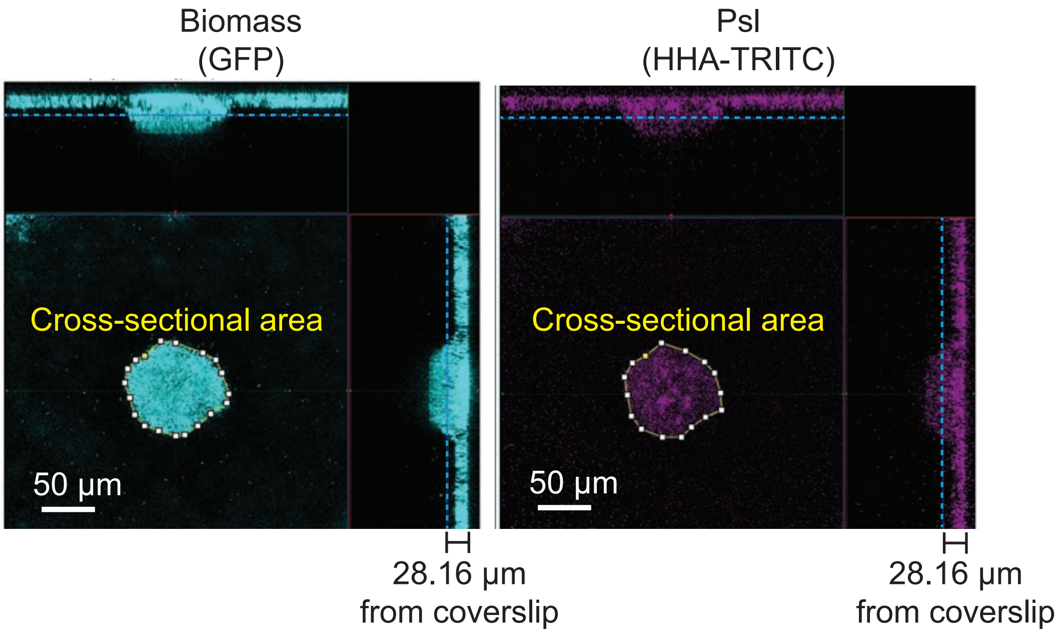

Supplemental Fig. 2. We measured the cross-sectional areas of biofilms that were pulse-chase labeled with fluorescently-conjugated Psl-specific lectins. For microscopy experiments of PAO1 flow cell biofilms, we determined changes in the cross-sectional areas of biomass (GFP) and Psl (HHA-TRITC or -Cy5). Images of the cross-section at 28.16 µm (72 to 86 h time-course) or 34.56 µm (96 to 108 h time-course) from the cover slip were identified using Zen imaging software (Zeiss) and exported. The cross-sectional area was measured in Fiji after spatial calibration by using the "polygon selections" tool to trace the circumference of the fluorescent area corresponding to either the biomass (GFP) or Psl (HHA-TRITC or -Cy5).

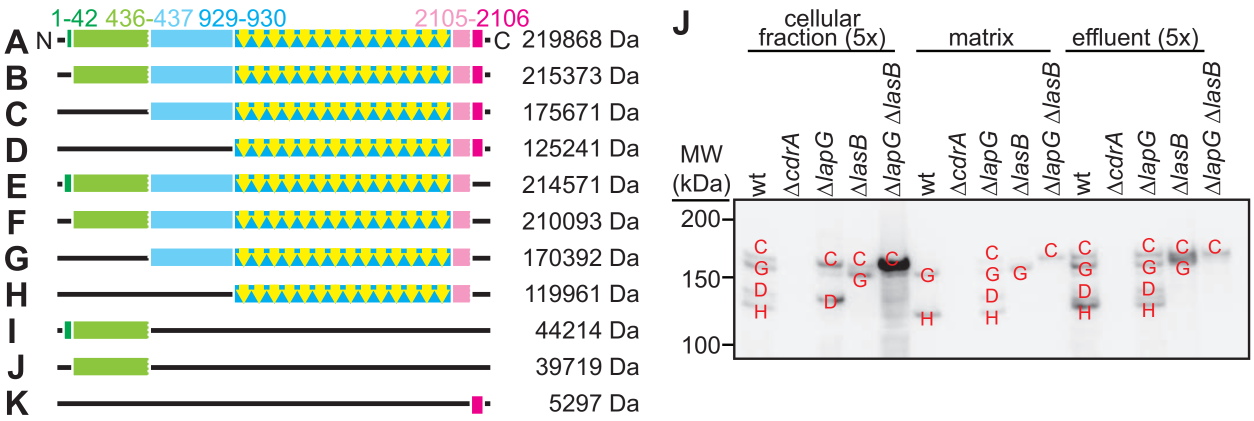

Supplemental Fig. 3. Anti-CdrA western blot analysis of biofilms supports that cell-free CdrA is primarily generated by LapG and the smaller CdrA fragments appear to be generated via proteolysis by LasB. (A-K) Schematic representations of CdrA and the possible structures and corresponding molecular weights that could result from processing. **(J)** The anti-CdrA western blot analysis of tube biofilm fractions of PAO1 and isogenic mutants from **Fig. 3** is reproduced here with possible CdrA structures that correspond to bands in the western blot indicated with red letters.

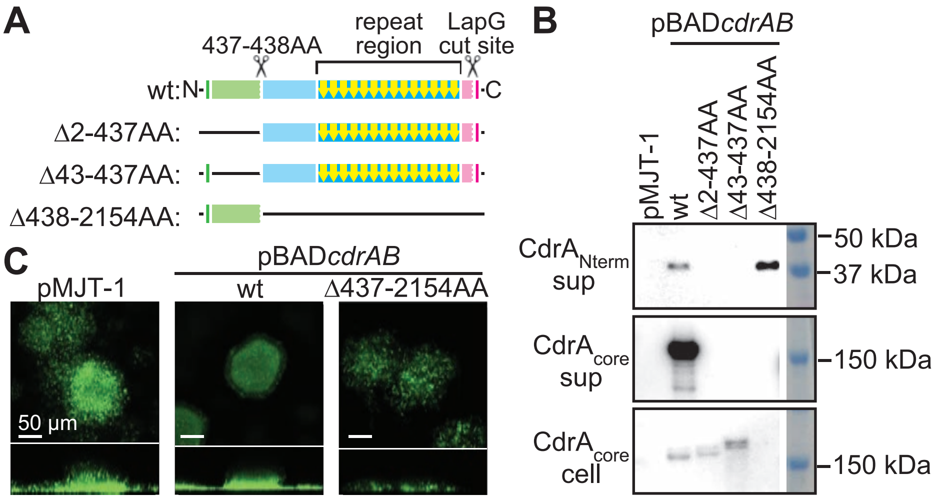

Supplemental Fig. 4. The N-terminal region of CdrA is necessary for secretion but alone does not promote biofilm formation. Previously, it was determined that the N-terminus of the approximately 150-kDa form of CdrA begins at residue 438[1]. However, the role of the N-terminal region of CdrA, which encompasses residues 1-437, was unknown. Homology modeling provided some clues about the function of this region. The first 42 amino acids were predicted to be a Sec-dependent secretion signal for export from the cytoplasm to the periplasm[1], and the remaining N-terminal region has homology to extended signal sequences in other adhesin proteins such as filamentous haemagglutinin (FHA) from *Bordetella pertussis* and HMW1 from *Haemophilus influenzae*[1–3]. Additionally, this N-terminal region has predicted structural motifs that include a hemagglutination site and sugar binding site[1]. Based on the structural predictions, we hypothesized that the N-terminal region may either (i) serve as an elongated signal sequence that is important for export of CdrA from the cell or (ii) promote bacterial aggregation/biofilm formation by binding to EPS. **(A)** Schematic representing CdrA in the different overexpression constructs that were engineered to study the role of the N-terminal region of CdrA. In the first, we deleted the entire N-terminal region, including the predicted Sec signal and extended signal sequence (Δ2-437AA) (theoretical molecular weight, 176 kDa). In the second, we deleted just the predicted extended signal sequence and left the Sec signal intact (Δ43-437AA) (theoretical molecular weight, 180 kDa). Finally, in the third, we deleted the entire C-terminal portion of the protein so that just the Sec signal and predicted extended signal sequence were present (Δ438-2154AA) (theoretical molecular weight, 44 kDa). These *cdrA* alleles were put on an arabinose-inducible vector, pMJT-1, along with *cdrB*. **(B)** We tested if the N-terminal region is required for export from the cell by performing western blot analysis of supernatant (sup) and cell preparations (cell) of PAO1Δ*cdrA* strains carrying these plasmids as well as the empty vector control pMJT-1 or pBAD*cdrAB,* carrying the wt *cdrA* allele. We used antibodies raised against peptides present in the core of CdrA ($\text{CdrA}_{\text{core}}$) and the N-terminal region of CdrA ($\text{CdrA}_{\text{Nterm}}$). Using the antibody specific for the N-terminal region of CdrA, we detected an approximately 40-kDa band in the supernatant fraction of the strain carrying the plasmid with the wild type (wt) *cdrA* allele, which corresponds to the theoretical molecular weight of the N-terminus, which is 44 kDa. Using the antibody specific for the CdrA-core, we detected bands corresponding to approximately 150-kDa in both the supernatant and cellular fractions of this strain. As expected, when the Sec signal was deleted (Δ2-437AA), we did not detect CdrA in the supernatant with either antibody but did detect the CdrA-core in the cellular fraction. If the Sec signal but not the extended signal domain was present (Δ43-437AA), we also did not detect CdrA in the supernatant, and again detected the CdrA-core in the cellular fraction.

Additionally, consistent with its predicted mass difference and the assumption that nothing is cleaved from the N-terminus of CdrA ∆43-437AA, the band from CdrA ∆43-437AA ran approximately 5-kDa higher than CdrA ∆2-437AA. Wt CdrA and CdrA ∆2-437AA migrated similarly in the gel, consistent with processing at the N-terminus of wt CdrA. The N-terminal region was found in the supernatant when only the N-terminal region was made (∆438-2154AA). These data supported that the entire N-terminal region of CdrA is required for export of CdrA from the cell. **(C)** Confocal microscopy images of flow cell biofilms of PAO1 ∆*cdrA* strains carrying the empty vector control, pMJT-1, or overexpression constructs that resulted in production of either wt CdrA or only the N-terminal region of CdrA (∆437-2154AA). The N-terminal region alone (∆437-2154AA) was unable to complement the *cdrA* phenotype. Together, these data suggest that the N-terminal region of CdrA does not promote biofilm formation, and support its function as an elongated signal sequence.

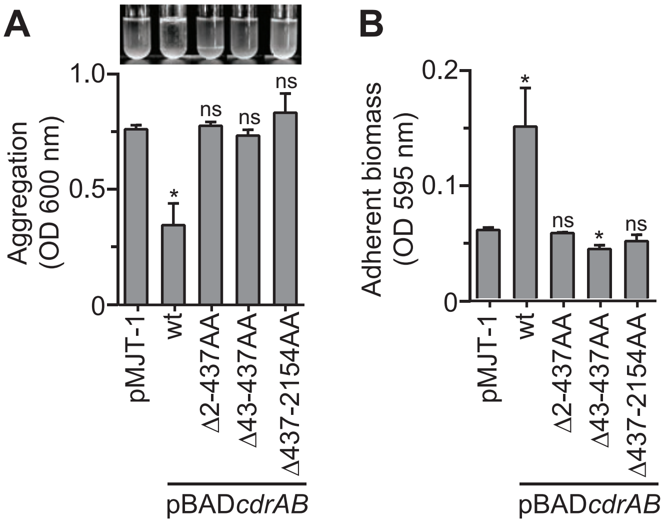

Supplemental Fig. 5. The N-terminal region of CdrA alone cannot mediate bacterial aggregation or static biofilm formation. Based on the predicted hemagglutination and sugar binding sites in the N-terminal region, we hypothesized that additionally it may bind to EPS to promote aggregation or biofilm formation[1]. It is important to note that the approximately 150-kDa form of CdrA lacking this N-terminal region has been shown to bind to Psl and Pel so the N-terminal region (up to 437 AA) was not expected to be the sole driver of CdrA-EPS interactions[1,4]. To test if this N-terminal region played a separate role in biofilm formation, we analyzed its contribution to liquid culture aggregation and static biofilm formation. **(A)** Optical density (OD 600 nm) of liquid cultures was measured to assess the aggregation of PAO1 Δ*cdrA* carrying plasmids for overexpression of truncated *cdrA* alleles as well as the empty vector control pMJT-1 plasmid and the plasmid carrying the wt *cdrA* allele. Liquid culture aggregation was only observed in the case of the plasmid carrying the wt *cdrA* allele, which is consistent with CdrA not being exported if the N-terminus is missing (and therefore not functional) and that the N-terminus alone is not sufficient to promote liquid culture aggregation. **(B)** These strains were also assessed for static biofilm formation using a crystal violet assay. Data represent the means of results from three replicates, and error bars indicate standard deviations. An asterisk indicates a significant difference compared to PAO1 Δ*cdrA* pMJT-1 (Student's *t* test; $P < 0.05$). n.s., not statistically significant.

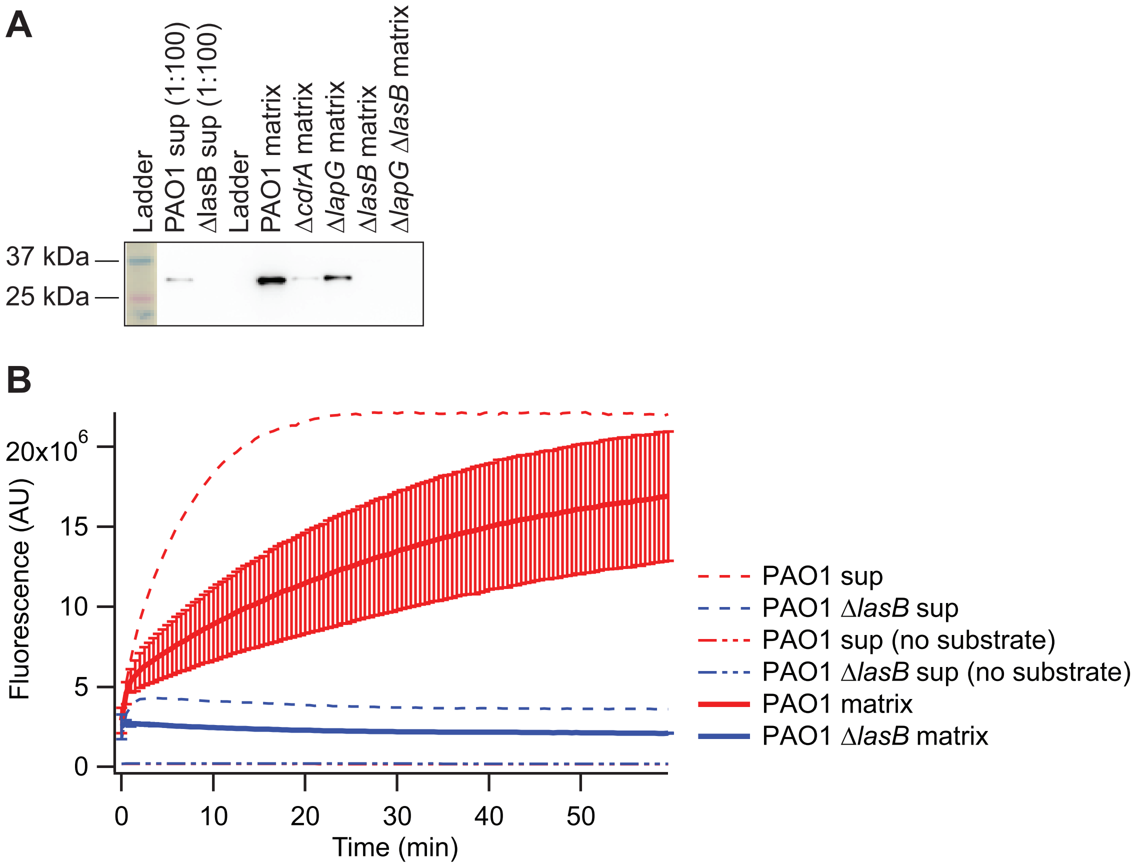

Supplemental Fig. 6. The extracellular protease LasB is retained in the biofilm matrix and is enzymatically active. **(A)** Anti-LasB western blot analysis of cell-free liquid culture supernatants (sup) and cell-free matrix extracted from tube biofilms. **(B)** Fluorogenic LasB activity assays of cell-free liquid culture supernatants (sup) and cell-free matrix extracted from tube biofilms. Data represent the means of results from three biological replicates that each contained three technical replicates, and error bars indicate standard deviations. Error bars are displayed for all conditions, but for some are quite small.

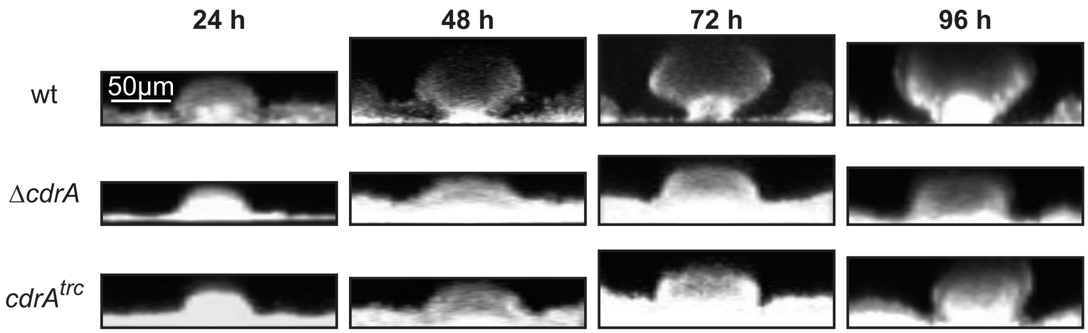

Supplemental Fig. 7. PAO1 Δ*wspF cdrA*^{trc}, aggregate growth is delayed relative to that of PAO1 Δ*wspF*. The formation of flow cell biofilm aggregates by PAO1 Δ*wspF*, PAO1 Δ*wspF* Δ*cdrA*, and PAO1 Δ*wspF cdrA*^{trc} was monitored over time by capturing confocal microscopy images at 24 h, 48 h, 72 h, and 96 h. Note that the images shown are not the same aggregates at each time point.

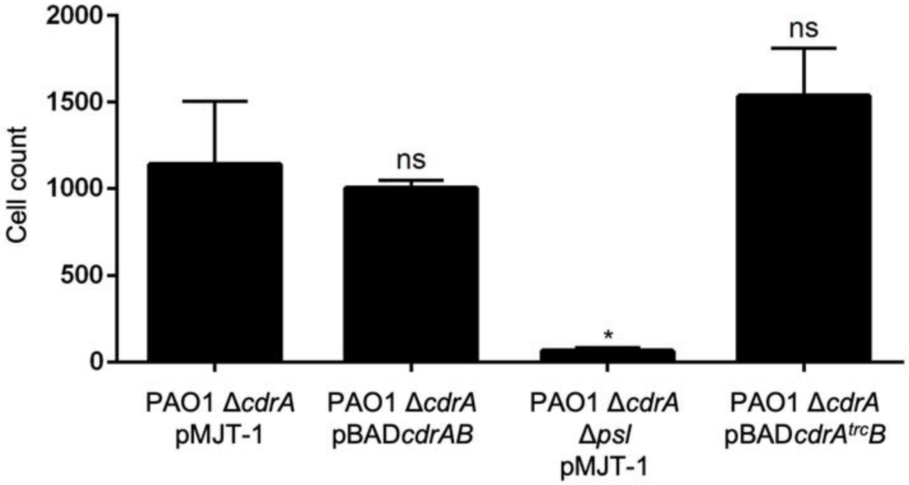

Supplemental Fig. 8. CdrA^{trc} does not cause a defect in initial attachment. The number of bacterial cells that attached to the coverslip following 20 min of media flow through the flow cell were determined. Data represent the means of results from 5 fields of view from each of three biological replicates, and error bars indicate standard deviations. An asterisk indicates a significant difference compared to PAO1 Δ*cdrA* pMJT-1 (Student's *t* test; $P < 0.05$), n.s., not statistically significant.

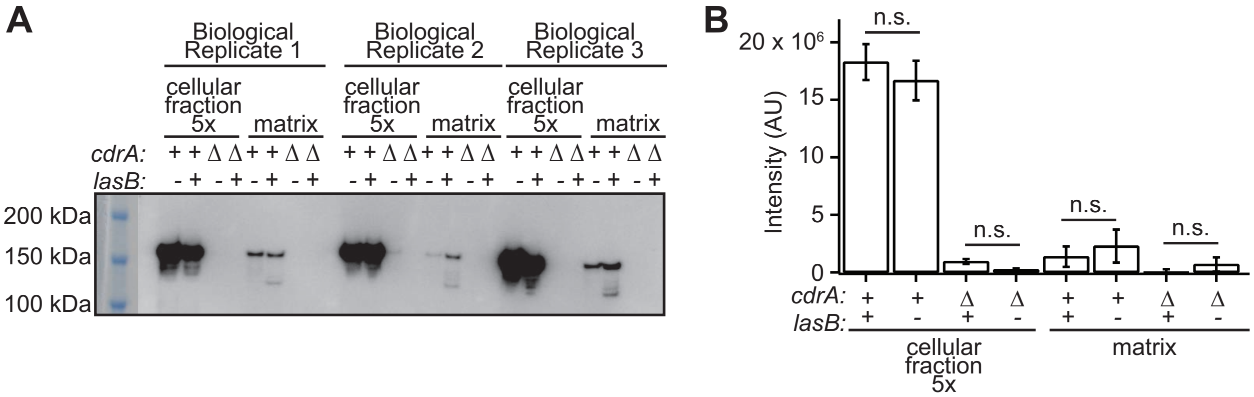

Supplemental Fig. 9. Overexpression of LasB does not decrease retention of CdrA in biofilms. (A) Anti-CdrA western blot analysis of cellular and cell-free matrix fractions extracted from tube biofilms in which we conditionally overexpressed *lasB* using a pBAD*lasB* plasmid in the PAO1 Δ*lasB* and PAO1 Δ*cdrA* Δ*lasB* strains. Biofilms grown with strains carrying the pBAD*lasB* plasmid are indicated as pBAD*lasB* "+" in the figure, and those with strains carrying the empty vector, pJN105, are indicated as pBAD*lasB* "-". The growth and extraction of tube biofilms was performed in biological triplicate. Note that this blot was cropped to show just Biological Replicate 1 and included in **Fig. 4C**. **(B)** Band intensities of the cellular and matrix fractions were determined using ImageJ. The band intensities at approximately 150-kDa and 125-kDa were summed. Error bars indicate standard deviation. (Student's *t* test; $P < 0.05$), n.s., not statistically significant.

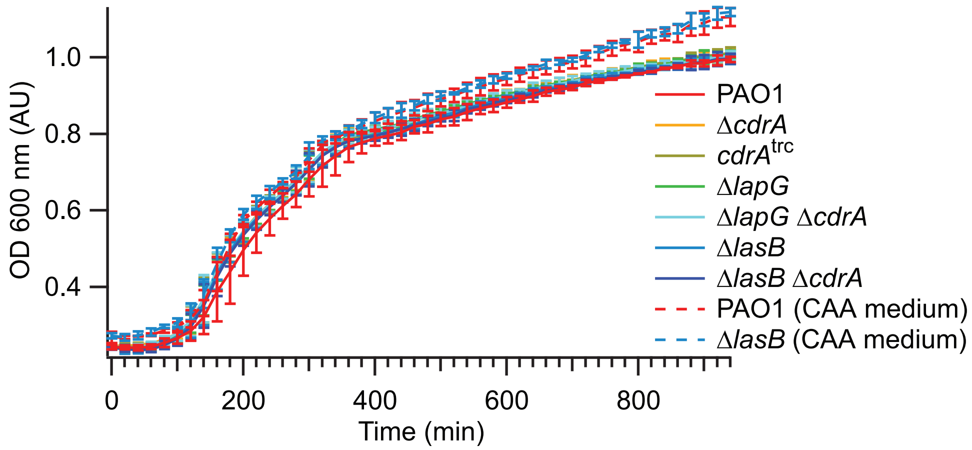

Supplemental Fig. 10. No LasB-dependent growth defect for planktonic cultures grown in either LB or casamino acid medium was observed. Growth curve of a panel of the isolates used in biofilm experiments. The isolates were grown in LB medium unless CAA medium is indicated. The data represent the means of results from 3 biological replicates that each contained 6 technical replicates, and error bars indicate standard deviations.

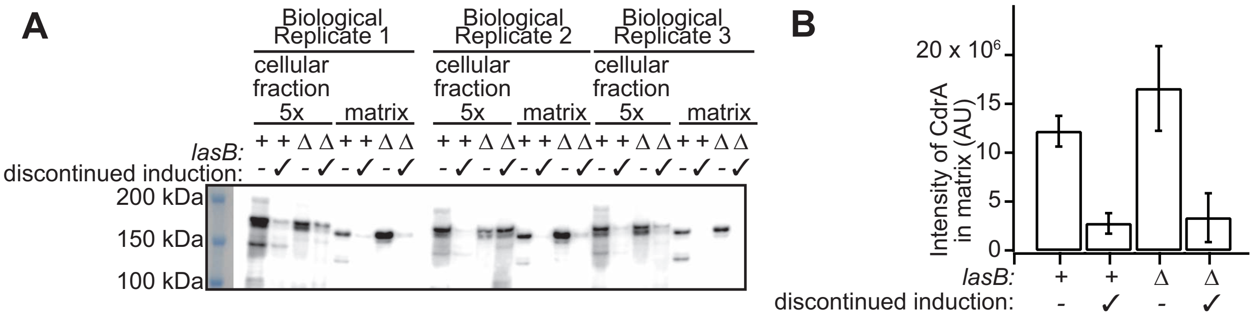

Supplemental Fig. 11. Continuous production of CdrA is not necessary for its retention in the biofilm matrix. (A) Anti-CdrA western blot analysis of cellular and cell-free matrix fractions extracted from tube biofilms in which we conditionally overexpressed *cdrAB* using a pBAD*cdrAB* plasmid in the PAO1 Δ*cdrA* and PAO1 Δ*cdrA* Δ*lasB* strains for the first 48 h of growth. Then biofilms were either harvested at 48 h (-) or induction of *cdrAB* was discontinued for the subsequent 24 h and then the biofilms were harvested (indicated with a checkmark).The growth and extraction of tube biofilms was performed in biological triplicate. Note that this blot was cropped to show just Biological Replicate 1 and included in **Fig. 4D**. **(B)** Band intensities of the matrix fractions were determined using ImageJ. The band intensities at approximately 150-kDa and 125-kDa were summed to give the total intensities displayed in the plot. Error bars indicate standard deviation.

Supplemental Table 1. The molecular weights in kDa were calculated for the bands in the western blot shown in **Fig. 3** and **Supplemental Fig. 2.** The migration distances of the bands in the ladder were used to prepare a standard curve, which was then used to determine molecular weights of based on the migration distances of the sample bands.

**cellular fraction**			
**PAO1**	**Δ*lapG***	**Δ*lasB***	**Δ*lapG* Δ*lasB***
182.8	171.7	166.4	169.0
166.4	135.7	158.7	
144.4			
131.5			
**matrix fraction**			
**PAO1**	**Δ*lapG***	**Δ*lasB***	**Δ*lapG* Δ*lasB***
158.7	177.1	166.4	177.1
123.5	158.7		
	135.7		
	125.4		
**effluent fraction**			
**PAO1**	**Δ*lapG***	**Δ*lasB***	**Δ*lapG* Δ*lasB***
179.9	182.8	185.7	179.9
169.0	169.0	177.1	
129.4	144.4		
	131.5		

Supplemental Table 2. Strains, plasmids, and primers used in this study.

Supplemental Table 2. Strains, plasmids and primers used in this study.

***P. aeruginosa* strains**		**Reference**
PAO1	wild-type	Holloway, 1955
PAO1 Δ*cdrA*	*cdrA* nonpolar mutation	Reichhardt, 2018
PAO1 Δ*lapG*	*lapG* nonpolar mutation	Rybtke, 2015
PAO1 Δ*lasB*	*lasB* nonpolar mutation	This study
PAO1 Δ*lapG* Δ*lasB*	*lapG lasB* nonpolar mutation	This study
PAO1 Δ*cdrA* Δ*lasB*	*cdrA lasB* nonpolar mutation	This study
PAO1 Δ*cdrA* Δ*psl* Δ*pel* Δ*algD*	*cdrA,* nonpolar mutation; *pslBCD,* polar mutation of *psl* operon; *pelA,* polar mutatation of the *pel* operon; *algD* nonpolar mutation	Reichhardt, 2018
PAO1 Δ*cdrA* Δ*psl*	*cdrA pslD* nonpolar mutation	Reichhardt, 2018
PAO1 *cdrA::cdrA^trc^*	chromosomal truncation of the native *cdrA* allele	This study
PAO1 Δ*wspF*	*wspF* nonpolar mutation	Borlee, 2010
PAO1 Δ*wspF* Δ*cdrA*	*wspF cdrA* nonpolar mutation	Borlee, 2010
PAO1 Δ*wspF cdrA::cdrA^trc^*	chromosomal truncation of the native *cdrA* allele and *wspF* nonpolar mutation	This study
PAO1 pBAD*psl*	chromosomal replacement of the native promoter with *araC*-P_{BAD} promoter	Armbruster, 2016

***E. coli* strains**		
DH5α pΔ*cdrA*	cloning strain carrying plasmid with the *cdrA* deletion allele	Reichhardt, 2018
DH5α pΔ*lapG*	cloning strain carrying plasmid with the *lapG* deletion allele	Rybtke, 2015
DH5α pΔ*lasB*	cloning strain carrying plasmid with the *lasB* deletion allele	This study
DH5α p*cdrA^trc^*	cloning strain carrying plasmid with the *cdrA* truncation allele	This study

**Primers**	
CR32_cdrA-seq	ATCGGCGGCGACTTCCA
CR70_cdrA-seq	TTGCCAGGCTGCCGTT
CR45_cdrA-seq	GGAGGCATGGTCGAGGAAAA
CR46_cdrA-seq	GTACAGTCCCTGGCAACTCC
CR103_lasB_EcoR1_F1	CCGGAATTCCGGGCAGCAGCGGATCGTCGG
CR91_lasB_R1	CGGGACCACCGAGCCTTGTTCAGTTCTCCTGGTTTTTCAG
CR92_lasB_F2	GCTCGGTGGTCCCGGCCGGCACT

CF104_lasB_XbaI_R2	CTAGTCTAGACTAGGCCAGGTACTCGCCTTGCGC	
CR41_cdrA-trc_F1	GGGGACAAGTTTGTACAAAAAAGCAGGCTCA TGGACTGGGCCTGGTCAGCGGT	
CR53_cdrA-trc_R1	GAGCAAGCAGGTCGGCTAACCCTTTTCGCAATGCGCAC TAT	
CR43_cdrA-trc_F2	CCCTTTTCGCAATGCGCACTAT	
CR44_cdrA-trc_R2	GGGGACCACTTTGTACAAGAAAGCTGGGTA TCGGCCAGGGACACCTGCTC	
lasB_F_EcoRI	ATCCGGGAATTCTGAACAAGATGAAGAAGGTTT	
lasB_R_XbaI	ATGGCC TCTAGAGAGCTTACAACGCGCTCG	
CR121_F1_XbaI	GCTGCAGGTCGACTCTAGAATAGGGAGATTTTCATG	
CR123_R2_KpnI	GCGGCGATCAGCGCGGTACCGGCCTGCTGG	
CR124_D438_2154_F	GGCACCTGGCTGCTCGAC TAACCCTTTTCGCAATGC	
CR125_D438_2154_R	GCATTGCGAAAAGGGTTA GTCGAGCAGCCAGGTGCC	
CR128_D43_437_F	ATCAACGCGGCCATGGCG CCGACCACCCTGCGCATC	
CR129_D43_437_R	GATGCGCAGGGTGGTCGG CGCCATGGCCGCGTTGAT	
CR134_D2_437_F	AATAGGGAGATTTTCATG CCGACCACCCTGCGCATC	
CR136_R_EcoRI	TATGACCATGATTACGAATTCTCAGAAGCGCGCCACCAC GTTGAACAGG	
BB08_pBADcdrAB_XbaI	TATCTAGAATAGGGAGATTTTCATGGTCCGTCCG	
BB09_pBADcdrAB_EcoRI	TAGATTTCTCAGAAGCGCGCCACCACGTTGAACAGG	

**Plasmids**		
pΔ*cdrA*	*cdrA* deletion vector	Reichhardt, 2018
pΔ*lapG*	*lapG* deletion vector	Rybtke, 2015
pΔ*lasB*	*lasB* deletion vector	This study
p*cdrA*^{trc}	*cdrA* truncation vector	This study
pEX18Gm	Allelic exchange vector	Hoang, 1998
pMJT-1	*ara*C-P_{BAD} cassette of pJN105 cloned in pUC18	Kaneko, 2007
pBAD*cdrAB*	*cdrAB* overexpression plasmid	Borlee, 2010
pBAD*cdrAB* (*cdrA*^{trc})	*cdrA*^{trc} *cdrB* overexpression plasmid	This study
pJN105	empty vector control plasmid	Newman & Fuqua, 1999
pJN105-*lasB*	*lasB* overexpression plasmid	This study
pBAD*cdrAB* (Δ2-437AA)	*cdrA*(Δ2-437AA)*cdrB* overexpression plasmid	This study
pBAD*cdrAB* (Δ43-437AA)	*cdrA*(Δ43-437AA)*cdrB* overexpression plasmid	This study
pBAD*cdrAB* (Δ438-2154AA)	*cdrA*(Δ438-2154AA)*cdrB* overexpression plasmid	This study

References

1. Borlee, B. R. *et al. Pseudomonas aeruginosa* uses a cyclic-di-GMP-regulated adhesin to reinforce the biofilm extracellular matrix. *Mol Microbiol* **75**, 827–842 (2010).
2. Grass, S. & St. Geme III, J. W. Maturation and secretion of the non-typable *Haemophilus influenzae* HMW1 adhesin: roles of the N-terminal and C-terminal domains. *Mol Microbiol* **36**, 55–67 (2000).
3. Chevalier, N. *et al.* Membrane targeting of a bacterial virulence factor harbouring an extended signal peptide. *Microb Physiol* **8**, 7–18 (2004).
4. Reichhardt, C. *et al.* The versatile *Pseudomonas aeruginosa* biofilm matrix protein CdrA promotes aggregation through different extracellular exopolysaccharide interactions. *J Bacteriol* **202**, (2020).
